# Classical neurofibroma

**DOI:** 10.11604/pamj.2021.39.146.30169

**Published:** 2021-06-23

**Authors:** Pallavi Lalchand Harjpal, Rakesh Krishna Kovela

**Affiliations:** 1 Department of Neurophysiotherapy, Ravi Nair Physiotherapy College, Datta Meghe Institute of Medical Sciences, Sawangi, Meghe, Wardha, Maharashtra, India

**Keywords:** Neurofibroma, spinal cord tumors, schwannoma

## Image in medicine

We are presenting to you an magnetic resonance imaging (MRI) finding of a 27-year-old male who presented to us with complains of gradually progressing pain over the neck along with radiation to the upper extremity with more involvement of the right side than the left. He also complained of insidious onset of weakness in the upper extremity. Magnetic resonance imaging findings revealed T2 hyperintense mass (red arrow) in the intradural extramedullary mass involving the C3-C4 cervical vertebrae and the intervertebral disc in between, highly suggestive of neurofibroma. Spinal neurofibromas are frequently observed over the cervical cord and are usually asymptomatic. Our patient showed mild symptoms, probable reasons might be the slow growth rate of neurofibroma. He was referred for physiotherapy for further opinion. On physical examination, he had weakness in upper limb movement (MRC grade 2+) and weak grip strength. Surgery was the treatment of choice for him and he underwent surgical excision of it along with pre-operative physiotherapy management for 5 days. Post-surgery, there was an enormous improvement in his symptoms i.e. residual weakness persisting without any pain. He was prescribed strengthening and gripping exercises. Before discharge, he had a grade of 4+ on the MRC grading scale. Early medical and surgical management along with pre- and post-operative rehabilitation is the road to recovery in such patients.

**Figure 1:**
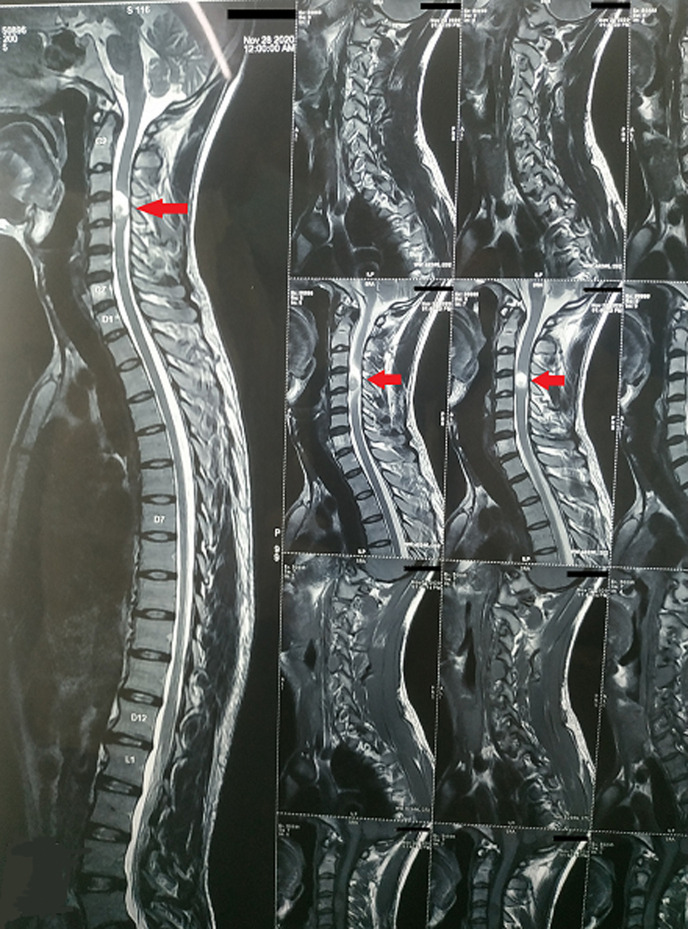
MRI findings of classical neurofibroma at the C3-C4 level

